# Enterococcus faecium pneumonia diagnosed by metagenomic next-generation sequencing in a patient with chronic obstructive pulmonary disease

**DOI:** 10.1016/j.idcr.2026.e02695

**Published:** 2026-07-21

**Authors:** Lidan Li, Danyang Wang, Chujun Huang

**Affiliations:** Department of Internal Medicine, Qujing Central Hospital, Qujing, China

**Keywords:** Enterococcus faecium pneumonia, Metagenomic next-generation sequencing, Chronic obstructive pulmonary disease, Community-acquired pneumonia, Culture-negative pneumonia, Opportunistic pathogen

## Abstract

*Enterococcus faecium* is a rare pathogen in community-acquired pneumonia (CAP), and its diagnosis is challenging, particularly when prior antibiotic therapy hampers isolation by conventional culture. We report a 72-year-old man with a 6-year history of chronic obstructive pulmonary disease (COPD) who presented with fever, cough, and progressive dyspnea. Despite empirical broad-spectrum antibiotics (piperacillin-tazobactam followed by imipenem-cilastatin), his condition deteriorated into acute respiratory distress syndrome (ARDS) requiring invasive mechanical ventilation. All routine cultures of blood, sputum, and throat swabs were negative, and extensive atypical pathogen screening was unrevealing. Metagenomic next-generation sequencing (mNGS) of sputum and subsequently bronchoalveolar lavage (BAL) fluid, performed at the ISO 15189-accredited central laboratory of Qujing Central Hospital of Yunnan Province, detected high read counts of *E. faecium* (sputum: 22,710 reads; BAL: 7921 reads; opportunistic pathogen, classification B), with simultaneous routine screening for 31 resistance genes, 4 resistance loci, and virulence genes, all negative. Additionally, sputum mNGS detected Epstein-Barr virus (Human gammaherpesvirus 4, 6715 reads, normal microbiota, classification C), and BAL fluid mNGS detected HSV-1 (Human alphaherpesvirus 1, 407 reads, normal microbiota, classification C). Both herpesviruses had classification C and were interpreted as non-pathogenic "bystanders"; no antiviral therapy was administered. The patient gradually improved on imipenem-cilastatin plus moxifloxacin and was successfully extubated and discharged. This case suggests that *E. faecium* can cause severe CAP in COPD patients, and mNGS is a valuable diagnostic tool when conventional cultures are negative; herpesviruses with classification C detected by mNGS should not be overinterpreted.

## Highlights

• E. faecium as a rare pathogen in severe COPD pneumonia, classified as opportunistic (class B).
• High mNGS reads vs. negative cultures for E. faecium, likely due to prior antibiotics.
• mNGS aids diagnosis when cultures are negative and screens for resistance/virulence genes.
• Respiratory HSV-1/EBV with classification C (normal flora) should not be overinterpreted.

## Introduction

Enterococci are Gram-positive cocci that normally colonize the human gastrointestinal tract. Enterococcus faecalis and Enterococcus faecium are important healthcare-associated pathogens causing urinary tract infections, bloodstream infections, infective endocarditis, and surgical site infections, but their role in lower respiratory tract infections is uncommon and controversial [1], [2], [3].

*E. faecium* has become a globally disseminated healthcare-associated pathogen due to its intrinsic ability to acquire antibiotic resistance [4]. A literature review found that most enterococcal pleuropulmonary infections occurred in elderly patients with comorbidities including immunosuppression, malignancy, renal failure, cardiovascular disease, or COPD [5]. However, community-acquired *E. faecium* pneumonia is even rarer [6]. Moreover, diagnosis is often delayed or missed—not because the organism is fastidious (enterococci grow readily on routine media)—but because conventional cultures may fail to isolate it from patients after antibiotic administration [7].

Metagenomic next-generation sequencing (mNGS) has emerged as a powerful tool for pathogen detection, especially when conventional methods yield negative results. By directly sequencing microbial DNA, mNGS can identify pathogens without prespecified targets, independent of microbial viability [7], [8], [9]. Furthermore, mNGS data can be simultaneously used for screening resistance genes and virulence factors. However, mNGS often detects multiple microorganisms simultaneously, requiring differentiation between primary pathogens and "bystanders" based on pathogenicity classification, read counts, and clinical context. We report a case of severe community-acquired *E. faecium* pneumonia diagnosed by mNGS, with concurrent detection of HSV−1 and EBV (classification C) determined to be non-pathogenic "bystanders.".

## Case report

A 72-year-old man presented with a 7-day history of cough, sputum production, wheezing, and fever. He had a 6-year history of COPD; pulmonary function testing 6 years earlier showed FEV₁/FVC < 0.7 and FEV₁ 58% of predicted, consistent with GOLD stage 2 COPD. His baseline functional status was dyspnea on climbing two flights of stairs (mMRC grade 2). In the past year, he had two outpatient acute exacerbations and one hospitalization 3 months prior (treated with piperacillin-tazobactam for 7 days). He had not used antibiotics or corticosteroids before this illness and had not received influenza or pneumococcal vaccines. He had a 30-pack-year smoking history but had quit 2 years ago.

Physical examination on admission: temperature 37.6 °C, pulse 86/min, respirations 20/min, blood pressure 145/81 mmHg, oxygen saturation 79% on room air. Chest examination revealed a barrel-shaped chest, diminished breath sounds, and scattered crackles.

Hospital day 1: Arterial blood gas on nasal cannula (FiO₂ 40%): pH 7.417, PaCO₂ 36.4 mmHg, PaO₂ 59.9 mmHg, PaO₂/FiO₂ ratio 150. Chest CT showed bilateral diffuse infiltrates and emphysema (Fig. 1). Laboratory tests (performed at the ISO 15189-accredited central laboratory of Qujing Central Hospital of Yunnan Province): leukocytosis (WBC 14.6 ×10⁹/L, neutrophils 89.7%), elevated CRP (91.2 mg/L). Blood, sputum, and throat swab cultures were obtained. A clinical diagnosis of acute exacerbation of COPD with lower respiratory tract infection and chronic respiratory failure was made. Empiric intravenous piperacillin-tazobactam (4.5 g q8h) was initiated.

**Fig. 1:**
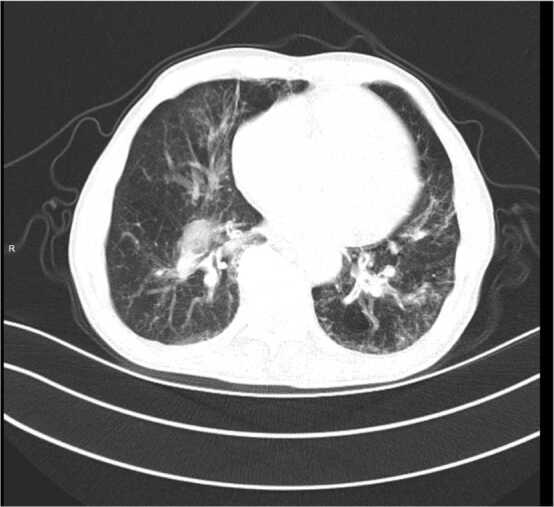
Chest CT on admission showing bilateral pulmonary infiltrates and emphysema.

Hospital day 3: ABG on face mask: pH 7.310, PaCO₂ 53.0 mmHg, PaO₂ 81.0 mmHg, PaO₂/FiO₂ ratio 202.5. Laboratory: WBC 14.9 × 10⁹/L, neutrophils 96.6%, CRP 217.3 mg/L, procalcitonin 0.572 µg/L. Chest CT showed progression of bilateral infiltrates and a new small pleural effusion (Fig. 2). All initial cultures were negative. Extensive atypical pathogen screening was unrevealing. Despite improved oxygenation, the patient remained febrile with hemoptysis and worsening inflammatory markers. After multidisciplinary consultation, antibiotics were changed to imipenem-cilastatin (1.0 g q8h), and blood and sputum samples were sent for mNGS (performed at the central laboratory of Qujing Central Hospital of Yunnan Province).

**Fig. 2:**
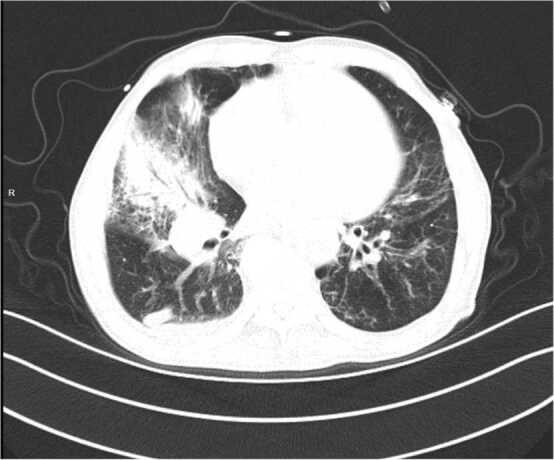
Chest CT on day 3 of hospitalization showing progressive bilateral pulmonary infiltrates and a new small right-sided pleural effusion.

Hospital day 5: The patient’s respiratory status deteriorated with confusion. He was transferred to the ICU and diagnosed with ARDS (meeting Berlin criteria). Endotracheal intubation and mechanical ventilation were initiated. Antibiotics were escalated to imipenem-cilastatin plus moxifloxacin (0.4 g qd). BAL was performed, and fluid was sent for culture and mNGS.

Hospital day 7: Blood mNGS was negative. Sputum mNGS detected:

*E. faecium* (22,710 reads, concentration 1.32 ×10⁹ copies/mL, opportunistic pathogen, classification B).

EBV (Human gammaherpesvirus 4, 6715 reads, normal microbiota, classification C)（Table 1）.

**Table 1:** Microorganisms detected by mNGS in blood, sputum, and BAL fluid samples.

Specimen	Microorganism	Latin name	Type	Reads	Concentration (copies/mL)	Pathogenicity
Blood	Not detected	—	—	—	—	—
Sputum	*E. faecium*	Enterococcus faecium	Bacterium	22,710	1.32 × 10⁹	B
Sputum	EBV	Human gammaherpesvirus 4	DNA virus	6715	3.90 × 10⁸	C
BAL fluid	*E. faecium*	Enterococcus faecium	Bacterium	7921	1.50 × 10⁶	B
BAL fluid	HSV−1	Human alphaherpesvirus 1	DNA virus	407	7.69 × 10⁴	C

Note: Resistance gene, resistance locus, and virulence gene screening were all negative. Pathogenicity classification was based on our laboratory standards: Class A = pathogenic microorganisms; Class B = opportunistic pathogens; Class C = normal microbiota, generally not considered pathogenic.

Resistance gene, resistance locus, and virulence gene screening were all negative.

Hospital day 9: BAL fluid culture remained negative. BAL mNGS detected:

*E. faecium* (7921 reads, concentration 1.50 ×10⁶ copies/mL, classification B).

HSV−1 (Human alphaherpesvirus 1, 407 reads, classification C)（Table 1）.

Resistance gene screening remained negative. No susceptibility testing could be performed due to no viable growth. Both EBV and HSV−1 had classification C and were interpreted as non-pathogenic "bystanders"; no antiviral therapy was administered. The patient’s clinical condition began to improve (afebrile, improved oxygenation).

**Discharge:** After 5 days of mechanical ventilation, the patient was weaned and extubated on day 15, transferred back to the general ward, and discharged on day 21 in good condition. ABG on room air at discharge: pH 7.430, PaCO₂ 39.0 mmHg, PaO₂ 69.8 mmHg, PaO₂/FiO₂ ratio 332. Laboratory: WBC 6.6 × 10⁹/L, CRP 36.8 mg/L. At 1-month follow-up, the patient reported no fever, mild cough, and no limitation in daily activities.

## Microbiological methods

Conventional cultures were processed at the ISO 15189-accredited central laboratory of Qujing Central Hospital of Yunnan Province. Blood cultures were incubated in the BACT/ALERT® 3D system (bioMérieux) for 5 days. Sputum and BAL fluid specimens were inoculated onto blood agar, chocolate agar, and MacConkey agar, and incubated at 35 °C in 5% CO₂ for 72 h. BAL fluid was also inoculated onto anaerobic blood agar and incubated anaerobically for 72 h. Gram staining of BAL fluid showed abundant polymorphonuclear leukocytes with no clearly visible microorganisms.

mNGS was performed at the same ISO 15189-accredited central laboratory of Qujing Central Hospital of Yunnan Province, using the Illumina NextSeq 550 platform. Nucleic acid was extracted using the QIAamp DNA Microbiome Kit (Qiagen). Libraries were prepared using the Nextera XT DNA Library Preparation Kit (Illumina) and sequenced with a depth of approximately 20 million single-end reads (75 bp). Raw data were quality-filtered, human sequences were subtracted, and the remaining microbial sequences were aligned to a curated pathogen database using a proprietary bioinformatics pipeline. A species was considered detected if it had > 3 unique reads and a relative abundance > 0.1% after background subtraction. Pathogenicity classification was based on our laboratory standards: Class A = pathogenic microorganisms; Class B = opportunistic pathogens; Class C = normal microbiota.

The mNGS data were simultaneously analyzed for resistance genes, resistance loci, and virulence genes. Resistance gene screening covered 31 clinically important resistance genes, including carbapenemase genes (IMP, NDM, SPM, KPC, PER, VIM, SIM, DIM, OXA, ACT, GIM), ESBL genes (CTX-M, GES, VEB, CMY, OKP, TLA, ACC, MOX, MIR, SHV, FOX), quinolone resistance genes (QnrA, QnrB), methicillin resistance gene (mecA), colistin resistance gene (MCR), vancomycin resistance genes (vanA, vanB, vanC), and sulfonamide resistance gene (sul). Resistance locus screening covered 4 key loci of the Mycoplasma pneumoniae 23S rRNA gene (positions 2063, 2064, 2067, 2617). Virulence gene screening covered rmpA, fyuA, fepA, iroN, iutA, cnf1, sat, eta, etb, tox, and MPN372.

## Discussion

This case report describes severe community-acquired *E. faecium* pneumonia in a patient with underlying COPD, diagnosed by mNGS after multiple negative conventional cultures. Several aspects warrant discussion: the rarity of *E. faecium* as a pulmonary pathogen, the diagnostic challenges posed by negative cultures in the context of antibiotic exposure, the role of mNGS in overcoming these limitations, and the clinical interpretation of mNGS results according to pathogenicity classification.

*E. faecium* is increasingly recognized as an important healthcare-associated pathogen, particularly in immunocompromised hosts, patients with prolonged hospitalization, or prior antibiotic exposure [1], [4]. The *E. faecium* clonal complex 17 (CC17) is a leading global cause of healthcare-acquired infections [2]. However, enterococcal pneumonia remains rare. Li et al. found that most enterococcal pleuropulmonary infections occurred in elderly patients with comorbidities including cardiovascular disease, chronic kidney disease, malignancy, or structural lung disease [5]. Portero et al. reported the first case of community-acquired *E. faecium* pneumonia in 1994, and subsequent reports remain extremely limited [6]. The unique aspect of our case is that the patient had community-acquired infection with COPD but no recent hospitalization or healthcare exposure.

The most striking diagnostic feature is the persistent failure of conventional culture to isolate *E. faecium* despite high microbial loads detected by mNGS. This discrepancy is not because enterococci are fastidious—on the contrary, *E. faecium* grows readily on standard media such as blood agar. The reasons for persistently negative conventional cultures are likely multifactorial. Firstly, although the patient denied antibiotic use prior to admission, unrecognized or unreported antimicrobial exposure during the 7-day symptomatic period cannot be excluded, as patients often overlook over-the-counter medications or treatments administered at primary care clinics. Secondly, specimens were obtained after empiric broad-spectrum antibiotics were initiated on hospital day 1, and BAL fluid was collected after 5 days of continuous antimicrobial exposure, which may have suppressed bacterial viability while leaving sufficient microbial DNA for mNGS detection [7]. Lastly, *E. faecium* is an infrequent pathogen in the lower respiratory tract and may have been present in low bacterial loads, further complicating isolation by conventional methods. mNGS detects DNA from both viable and non-viable organisms, making it particularly useful in the setting of antibiotic pretreatment.

The cost of mNGS testing at Qujing Central Hospital of Yunnan Province is 600 RMB per sample, substantially lower than the reported market price (approximately 3000–4000 RMB), significantly enhancing clinical accessibility.

In this case, mNGS screening for 31 resistance genes, 4 resistance loci, and virulence genes was all negative. The negative resistance gene results have important clinical implications: negative vanA, vanB, vanC, and carbapenemase genes suggest that the *E. faecium* isolate may remain susceptible to vancomycin and carbapenems, partly explaining the favorable clinical response to imipenem-cilastatin plus moxifloxacin. Negative virulence gene screening suggests the strain’s virulence may be limited, and the severe presentation may be more attributable to underlying COPD-related lung damage.

Of particular note are the herpesviruses detected: EBV (6715 reads) in sputum and HSV−1 (407 reads) in BAL fluid. Both had pathogenicity classification C (normal microbiota). This classification has important clinical implications. According to our laboratory standards, Class C represents normal microbiota, generally not considered pathogenic. EBV and HSV−1 have extremely high global infection rates and can be detected in respiratory specimens as "bystanders" representing latent infection reactivation or asymptomatic shedding [10], [11]. Although HSV−1 and EBV can cause severe disease in immunocompromised patients, in most cases their detection in respiratory specimens represents colonization or shedding, not active infection [12]. In this case, the patient’s presentation (high fever, purulent sputum, markedly elevated CRP/procalcitonin) was more consistent with bacterial pneumonia; clinical and inflammatory markers improved with antibacterial therapy; there were no typical viral pneumonia imaging findings; and the patient recovered without antiviral therapy. This case suggests that mNGS results must be interpreted in conjunction with pathogenicity classification and clinical context—microorganisms with Class C (normal microbiota), even with substantial read counts, should not be readily identified as pathogens; while Class B opportunistic pathogens require comprehensive assessment based on host immune status and clinical evidence.

The diagnostic superiority of mNGS in post-antibiotic infections is increasingly recognized. Recent studies have confirmed that in immunocompromised patients with severe pneumonia, mNGS yields significantly higher positivity rates than conventional testing [8], [9]. Notably, for critically ill pneumonia patients in the ICU, mNGS had significantly higher detection rates for *E. faecium* and Pneumocystis jirovecii compared to conventional methods (*p* < 0.05) [9].

However, mNGS has limitations. First, despite reduced costs, widespread adoption remains constrained by equipment and personnel requirements. Second, respiratory specimens frequently contain nucleic acids from multiple colonizing organisms—the EBV and HSV−1 detected in this case are typical examples. Third, background microbial DNA from reagents or the environment can introduce noise, potentially causing false-positive results. Fourth, mNGS sensitivity for resistance gene detection depends on database coverage and sequencing depth, with possible missed low-abundance resistance genes.

This case has several limitations. First, no viable isolate was obtained for susceptibility testing. Second, the diagnosis of *E. faecium* pneumonia was based solely on mNGS results without culture confirmation; although high read counts strongly support a pathogenic role, colonization cannot be entirely excluded. Third, pathogenicity assessment of EBV and HSV−1 was based on classification C and clinical evaluation, without antiviral treatment validation. Fourth, the patient declined follow-up, limiting long-term outcome data.

In conclusion, this case demonstrates that *E. faecium* can cause severe community-acquired pneumonia in patients with underlying COPD, and conventional cultures may fail to detect it due to antibiotic pretreatment. mNGS provides a valuable diagnostic tool in such challenging cases. For microorganisms with Class C detected by mNGS, clinicians should avoid overinterpretation and unnecessary therapy; for Class B opportunistic pathogens, comprehensive assessment based on host immune status and clinical context is required. Clinicians should consider *E. faecium* as a potential pathogen in patients with COPD, prior antibiotic exposure, and poor response to empirical broad-spectrum therapy, and should employ molecular diagnostic methods when conventional cultures are negative.

## CRediT authorship contribution statement

**Wang Dan yang:** Software, Methodology, Conceptualization. **Lidan Li:** Writing – original draft, Resources, Data curation. **Huang jun Chu:** Writing – review & editing, Methodology, Investigation, Formal analysis.

## Ethics statement and informed consent

This case report is a single case description and does not involve interventional research. Written informed consent was obtained from the patient for publication of this case report and accompanying images. This study was reviewed and granted an exemption by the Medical Ethics Committee of Qujing Central Hospital of Yunnan Province.

## Declaration of Competing Interest

The authors declare that they have no known competing financial interests or personal relationships that could have appeared to influence the work reported in this paper.
